# A synthesis of mercury research in the Southern Hemisphere, part 1: Natural processes

**DOI:** 10.1007/s13280-023-01832-5

**Published:** 2023-03-21

**Authors:** Larissa Schneider, Jenny A. Fisher, María C. Diéguez, Anne-Hélène Fostier, Jean R. D. Guimaraes, Joy J. Leaner, Robert Mason

**Affiliations:** 1 School of Culture, History and Language. Australian National University, Coombs Bld 9 Fellows Rd, Acton. Canberra, ACT 2601 Australia; 2 School of Earth, Atmospheric and Life Sciences, University of Wollongong, Northfields Avenue, Wollongong, NSW 2522 Australia; 3 Instituto de Investigaciones en Biodiversidad y Medioambiente (Consejo Nacional de Investigaciones Científicas y Técnicas-Universidad Nacional del Comahue), 1250 San Carlos de Bariloche (8400), Quintral Argentina; 4 Instituto de Química/Unicamp, Rua Josué de Castro, s/n – Cidade Universitária, Campinas, SP 13083-970 Brazil; 5 Lab. de Traçadores, Inst. de Biofísica, Bloco G, CCS (Centro de Ciências da Saúde), Av. Carlos Chagas Filho 373, Rio de Janeiro, Ilha do Fundão CEP 21941-902 Brazil; 6 Department of Environmental Affairs and Development Planning, Western Cape Government, 1 Dorp Street, Western Cape, Cape Town, 8001 South Africa; 7 Department of Marine Sciences, University of Connecticut, 1080 Shennecossett Road, Groton, CT 06340 USA

**Keywords:** Background, Geogenic sources, Litterfall, Methylation processes, Oceans, Soils

## Abstract

Recent studies demonstrate a short 3–6-month atmospheric lifetime for mercury (Hg). This implies Hg emissions are predominantly deposited within the same hemisphere in which they are emitted, thus placing increasing importance on considering Hg sources, sinks and impacts from a hemispheric perspective. In the absence of comprehensive Hg data from the Southern Hemisphere (SH), estimates and inventories for the SH have been drawn from data collected in the NH, with the assumption that the NH data are broadly applicable. In this paper, we centre the uniqueness of the SH in the context of natural biogeochemical Hg cycling, with focus on the midlatitudes and tropics. Due to its uniqueness, Antarctica warrants an exclusive review of its contribution to the biogeochemical cycling of Hg and is therefore excluded from this review. We identify and describe five key natural differences between the hemispheres that affect the biogeochemical cycling of Hg: biome heterogeneity, vegetation type, ocean area, methylation hotspot zones and occurence of volcanic activities. We review the current state of knowledge of SH Hg cycling within the context of each difference, as well as the key gaps that impede our understanding of natural Hg cycling in the SH. The differences demonstrate the limitations in using NH data to infer Hg processes and emissions in the SH.

**Supplementary Information:**

The online version contains supplementary material available at 10.1007/s13280-023-01832-5.

## Introduction

**Fig. 1:**
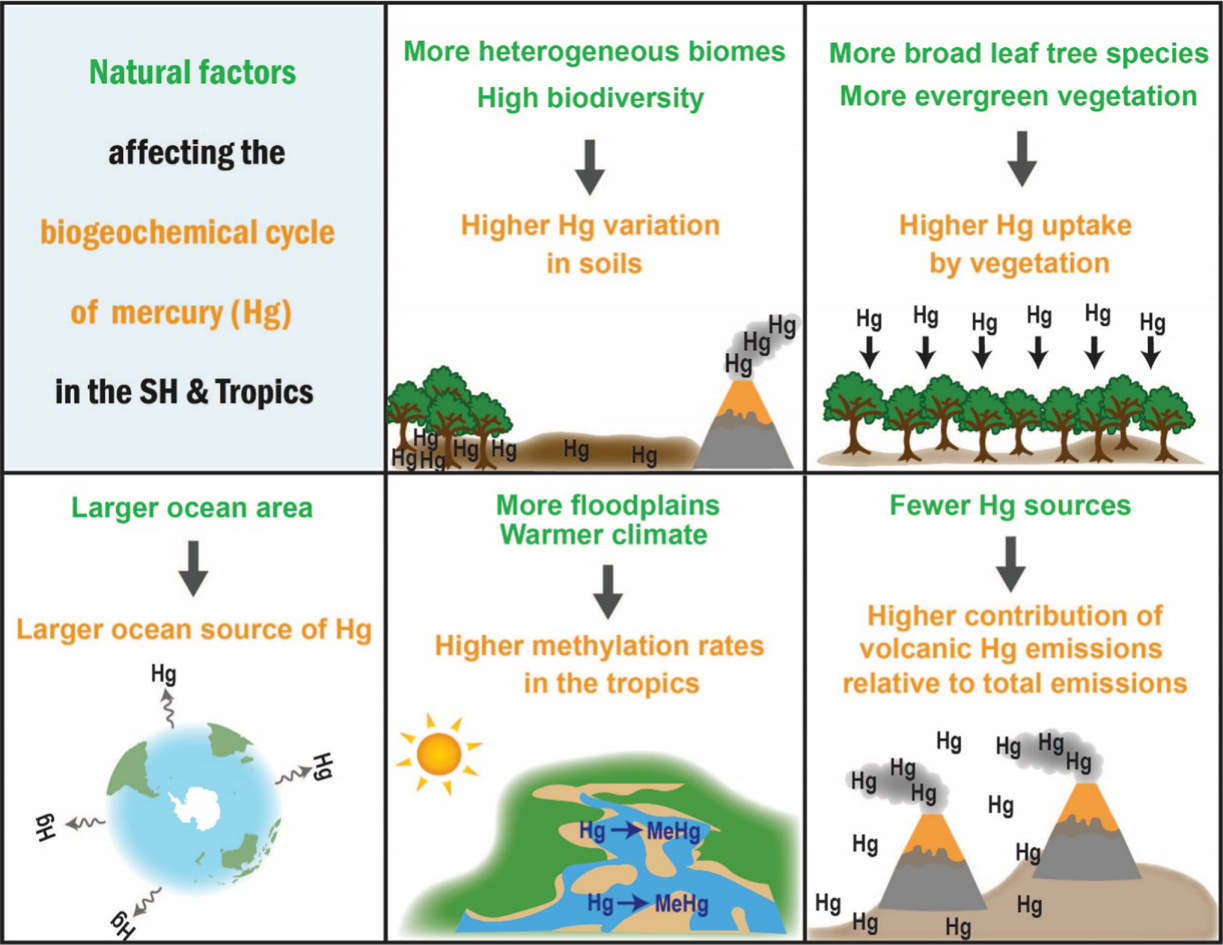
Conceptual overview of the key differences in the natural mercury (Hg) cycle in the Southern Hemisphere and Tropics (relative to the NH)

Over the past two decades, our understanding of the biogeochemical cycling of mercury (Hg) has advanced significantly, underpinned by a substantial growth in Hg research publications. The current global Hg cycle, as well as Hg regulation and policies, is based on an extensive body of scientific, empirical and modelling studies. However, the vast majority of this work is based on Hg data collected in the Northern Hemisphere (NH), which are mostly not representative of the unique conditions of the Southern Hemisphere (SH). In this paper, we identify and describe five key natural differences between the hemispheres that influence Hg sources, biogeochemical cycling and environmental impacts (Fig. 1). A companion paper (Fisher et al. 2023) focuses on the key human-influenced differences between hemispheres.

Historical consideration of the global nature of Hg pollution derived in part from the longer earlier estimated atmospheric lifetime of elemental Hg (Hg^0^) of 12–18 months (Lamborg et al. 2002; Seigneur et al. 2004; Lindberg et al. 2007; AMAP/UNEP 2008), similar to the timescale for interhemispheric air exchange (Geller et al. 1997; Kersting et al. 2020). However, more recent estimates suggest a shorter atmospheric lifetime of 3–6 months (Horowitz et al. 2017; Saiz-Lopez et al. 2020; Shah et al. 2021; Zhang and Zhang 2022). This shorter lifetime implies that Hg is largely a hemispheric pollutant (Corbitt et al. 2011; Driscoll et al. 2013), thus increasingly placing importance towards considering Hg sources, sinks and impacts from a hemispheric perspective.

In the SH, data available to constrain Hg cycling processes are limited. Many SH environments have only been sampled sporadically for Hg, if at all, and there are very few long-term Hg datasets available (Table 1). For example, the 2018 Global Mercury Assessment (GMA) identified only six sites with long-term (10 years or more) air monitoring data in the SH, compared to the 22 operating in the NH (UNEP 2019). Of these six SH sites, only three were continental (two in South America, one in Africa, none in Oceania) (Table 1); however, monitoring at the two South American sites has since ceased due to a lack of funding. Similarly, oceanographic cruises measuring Hg in the South Pacific, South Atlantic and around Antarctica are limited in scope compared to cruises undertaken in the NH. This problem is not unique to air and ocean monitoring of Hg: a 2016 global review of Hg monitoring networks identified data gaps across the SH for human and biota monitoring as well (UNEP 2016). In the absence of comprehensive Hg data from the SH, findings from the NH are generally assumed to be broadly applicable (Wang et al. 2016; Friedli et al. 2009; Agnan et al. 2016), often overlooking localized SH conditions. Geographic and environmental differences between the hemispheres mean that this knowledge cannot be easily translated from the NH to the SH.

**Table 1:** Long-term atmospheric Hg monitoring sites in the Southern Hemisphere

Site name	Region	Included in the Global Mercury Assessment 2018 (UNEP 2019)*	Reference
Manaus	South America	Yes	Sprovieri et al. (2016)
Bariloche	South America	Yes	Diéguez et al. (2019)
Cape Point	South Africa	Yes	Slemr et al. (2015, 2020), Martin et al. (2017)
Cape Grim**	Australia	No	Slemr et al. (2015)
Gunn Point	Australia	No	Howard et al. (2017)
Amsterdam Island	Indian Ocean	Yes	Angot et al. (2014), Slemr et al. (2015, 2020)
Dumont d’Urville	Antarctica	Yes	Angot et al. (2016a)
Concordia	Antarctica	Yes	Angot et al. (2016b)
Troll Research Station	Antarctica	No	Pfaffhuber et al. (2012), Slemr et al. (2015)

* Only six of the nine sites with air monitoring data in the Southern Hemisphere were included in the 2018 Global Mercury Assessment (UNEP 2019). This is because only these six sites had a long-term (10 years or more) time series at the time of publication

**Note that the data from this original publication has been corrected from mean of 0.85 ng m^−3^ to 0.93 ng m^−3^ as discussed in Fisher and Nelson (2020) and at https://www.mercury-australia.com.au/measuring-gaseous-elemental-mercury-in-regional-background-air/

Here, we identify and describe five natural differences between the hemispheres that have implications for Hg cycling (Fig. 1), which span a range of timescales and influence Hg processes occurring both within and between atmospheric, terrestrial, aquatic and biological reservoirs. We elaborate on each key difference between the hemispheres and review the current state of knowledge on SH Hg cycling within the context of each difference. The key gaps that impede our understanding of natural Hg cycling in the SH are also identified.

**Fig. 2:**
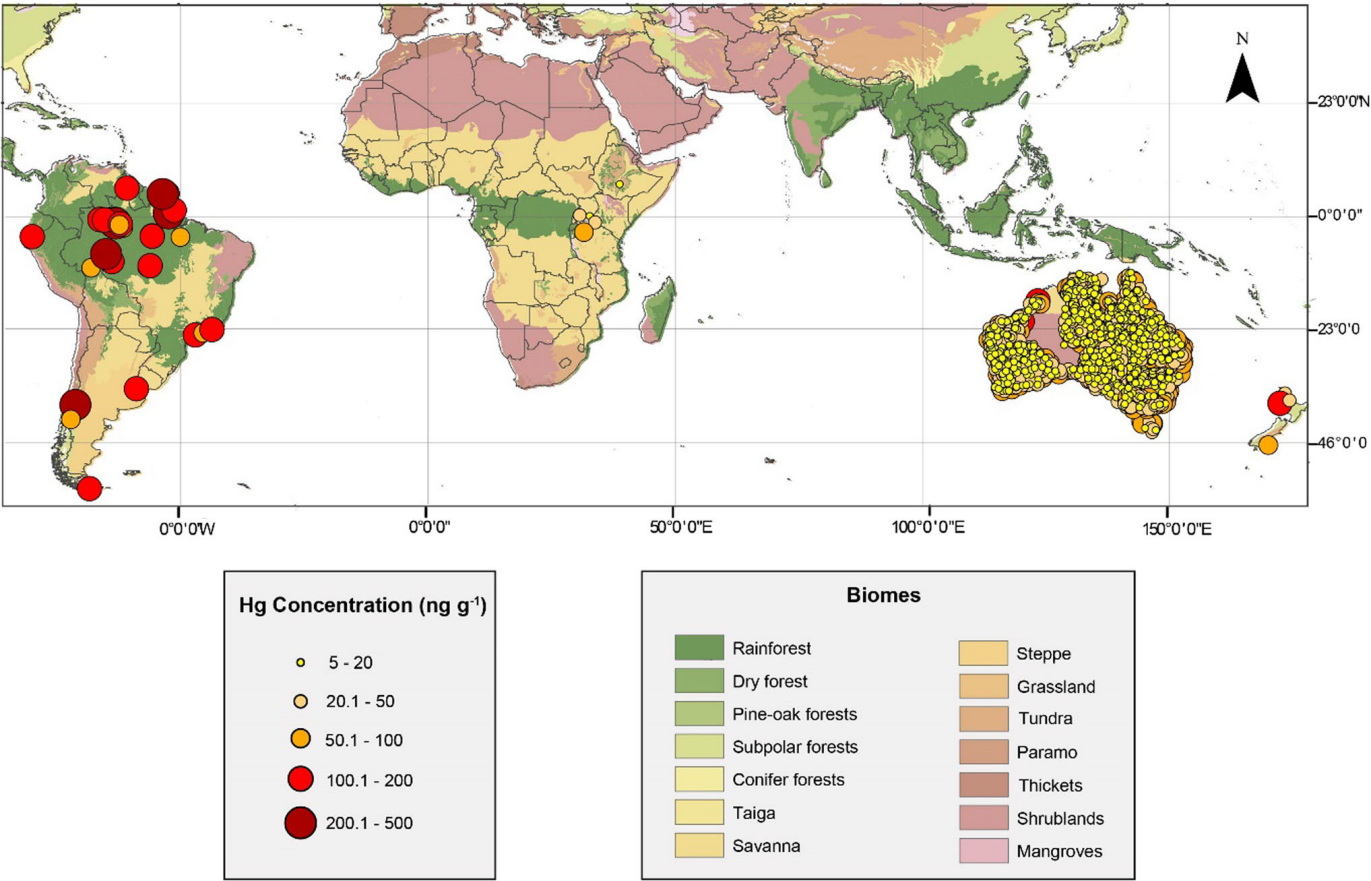
Mercury concentrations in uncontaminated soils (top five centimeters) of the Southern Hemisphere and Tropics. Data and sources of information are given in Tables 2 and S1. Basemap from the World Wildlife Fund, Terrestrial Ecosystems of the World, version 2.0 (Olson et al. 2001)

Our focus here is on the SH midlatitude and tropical regions (0–50°S), extending to the NH tropics (0–23.5°N) when relevant (Fig. 2). The Antarctic region is excluded from this review as its unique Hg cycle makes it difficult to compare with lower latitude regions. Antartica is pristine, impacted by different biological and physical processes (for example, snow and sea ice processes), and has a different effect on the earth’s climate and ocean systems. Hence, its contribution to the biogeochemical cycling of Hg justifies an exclusive review similar to that recently conducted for the Arctic (Dastoor et al. 2022).

**Table 2:** Mercury (Hg) concentrations (ng g^−1^) in non-contaminated soils in countries of the SH & Tropics. Please refer to Table S1 for references and a complete dataset for Australia. CVAFS: Cold vapour atomic fluorescence spectroscopy; CVAAS: Cold Vapor Atomic Absorption Spectroscopy; CVOES: cold vapor optical emission spectroscopy; ICPMS: Inductively coupled plasma mass spectrometry; INAA: Instrumental Neutron Activation Analysis; TDAAS: Thermo Desorption Atomic Absorption Spectroscopy

Country	Latitude	Longitude	Hg concentration (ng g^−1^)	Analytical method	References
Argentina	− 37.7833	− 70.9200	370	INAA	Perez Catán et al. (2020)
Argentina	− 40.7333	− 71.7700	75	INAA	Rizzo et al. (*submitted*)
Argentina	− 54.7167	− 68.0000	149	CVAAS	Peña-Rodríguez et al. (2014)
Argentina	− 34.4667	− 58.5000	198	CVAAS	Marbán et al. (1999)
Ecuador	− 3.7200	− 79.6200	200	CVAAS	Schudel et al. (2018)
French Guyana	4.8300	− 53.2500	300	CVAAS	Guedron et al. (2006)
French Guyana	5.0300	− 53.0500	550	CVAAS	Grimaldi et al. (2008)
French Guyana	4.9400	− 52.3300	199	CVAFS	Roulet and Lucotte (1995)
Brazil	− 3.6167	− 55.3200	137	CVAFS	Roulet et al. (1998)
Brazil	− 3.8181	− 49.6500	71	CVAAS	Aula et al. (1994)
Brazil	0.9200	− 52.0000	229	CVAFS	De Oliveira et al. (2001)
Brazil	1.5100	− 50.9100	113	CVAFS	Adler Miserendino et al. (2017)
Brazil	− 9.9444	− 56.3300	79	TDAAS	Richter et al. (*submitted*)
Brazil	− 8.7503	− 63.4600	148	TDAAS	Richter et al. (*submitted*)
Brazil	− 22.5344	− 42.8600	40	TDAAS	Buch et al. (2017)
Brazil	− 22.5978	− 43.2400	150	TDAAS	Buch et al. (2017)
Brazil	− 2.0169	− 61.1900	91	CVAAS	Fadini and Jardim (2001)
Brazil	− 0.9581	− 62.9100	231	CVAAS	Fadini and Jardim (2001)
Brazil	− 1.1017	− 62.7200	204	CVAAS	Fadini and Jardim (2001)
Brazil	− 0.3475	− 65.9100	132	CVAAS	Fadini and Jardim (2001)
Brazil	− 0.3708	− 65.0400	156	CVAAS	Fadini and Jardim (2001)
Brazil	− 1.2333	− 61.8300	156	CVAFS	Magarelli and Fostier (2005)
Brazil	− 1.3850	− 61.9700	84	CVAFS	Magarelli and Fostier (2005)
Brazil	− 10.0286	− 67.6800	84	CVAFS	Melendez-Perez et al. (2014)
Brazil	− 9.6333	− 55.7700	137	CVAAS	Lacerda et al. (2004)
Brazil	− 3.6667	− 55.3500	113	CVAFS	Patry et al. (2013)
Brazil	− 23.6500	− 46.6200	116	CVAAS	Fostier et al. (2003)
Brazil	− 23.2694	− 45.0481	87	CVAAS	Fostier et al. (2003)
Brazil	− 22.5978	− 43.2400	140	CVAAS	Buch et al. (2015)
New Zealand	− 35.4175	173.8519	41	CVAAS	Davey and Moort (1986)
New Zealand	− 37.4175	172.8519	155	CVAAS	Davey and Moort (1986)
New Zealand	− 45.8795	170.5006	82	ICPMS	Martin et al. (2017)
New Zealand	− 36.8509	174.7645	40	CVAAS	Auckland Regional Council. (2001)
Tanzania	6.7924	39.2083	20	CVAAS	Campbell et al. (2003)
Tanzania	− 2.8000	31.9666	80	CVAAS	Campbell et al.(2003)
Uganda	0.4003	33.2000	24	CVOES	Campbell et al. (2003)
Uganda	0.5056	31.1459	34	CVAAS	Campbell et al. (2003)
Uganda	− 0.9001	34.3090	42	CVAAS	Campbell et al. (2003)
Venezuela	6.0000	− 60.5000	120	CVAFS	Santos-Francés et al. (2011)
Australia	− 24.9900	151.4400	24	CVAFS	Rayment et al. (1997)
Australia	− 26.1100	153.3600	80	CVAFS	Rayment et al. (1998)
Australia	− 36.5000	148.2667	47	CVAFS	Packham et al. (2009)
Australia	− 32.0940	150.9890	18	CVAAS	Schneider (2021)
Australia	− 32.0481	151.0151	54	CVAAS	Schneider (2021)
Australia	Several locations		26 (5–160)	ICPMS	de Caritat and Cooper (2011)*

* The full Hg data from the National Geochemical Survey of Australia is available in Table S1

## Background mercury in soils

Natural (background) Hg concentrations are variable and are often the subject of debate as most ecosystems are greatly influenced by a long history of anthropogenic activities that impact surface soil concentrations (de Caritat and Cooper 2011; Reimann and de Caritat 2017; Lado et al. 2008). This is further complicated in the SH and tropics, where most Hg studies have focused on contaminated sites impacted by artisanal and small-scale gold mining (ASGM) and other point sources of contamination, with little information available on background Hg in soils (Guimaraes 2020). In the context of this synthesis paper, background soils are defined as “*soils collected at sites with no direct anthropogenic source of Hg.*”

Although soils may have a long-term regional signal of Hg inputs resulting from long-range Hg transport, geology and weathering processes are the dominant factors that influence Hg concentrations in soil. While studies in the NH have considered soils to be enriched in Hg when concentrations are > 100 ng g^−1^ (Gustin et al., 2000), the published data for the SH show a wider range of Hg concentrations in soils (Table 2) that makes it difficult to establish a firm threshold limit for background Hg in soils.

### Mercury distribution in podzols versus oxisols

The soil Hg distribution profile with depth is one of the main differences between the SH and NH. In the NH, Hg accumulation and distribution in podzol soils of boreal and temperate environments are mainly controlled by organic matter content (Schuster 1991; Hintelmann et al. 2002; Skyllberg et al. 2006), with the highest concentrations found in the uppermost soil horizons (Schwesig and Matzner 2000; Obrist et al. 2011). In equatorial South America (in remote forest oxisols), peaks of Hg have been reported for the subsurface soil (mineral layer of B horizons) and attributed to the downward translocation of Hg as a result of pedogenetic processes such as podzolization (Roulet et al. 1998; Grimaldi et al. 2008; Guedron et al. 2009).

The temperate and nordic soils of the NH have a thick organic layer enriched with Hg (Grigal et al. 1994; Grondin et al. 1995), while the heat, humidity and the paucity of nutrients in tropical soils cause rapid turnover of organic matter, depleting the topsoil layer of organic matter (Roulet et al. 1998). Furthermore, the ferralitic soils of dense tropical forests have the capacity to rapidly mineralise plant litter in the organic layer (Roulet et al. 1998). These factors hinder the formation of a thick organic layer in tropical soils, limiting superficial Hg accumulation, while promoting Hg absorption to sub-superficial mineral layers rich in oxy-hydroxide iron and aluminium (Roulet and Lucotte 1995; Roulet et al. 1998; Lechler et al. 2000; De Oliveira et al. 2001; Fadini and Jardim 2001). As a result, Hg concentration in the mineral horizon of equatorial South American soils is higher than in the top organic layer (Lechler et al. 2000; De Oliveira et al. 2001; Fadini and Jardim 2001; Guedron et al. 2009).

Although podzols/spodzols soils are mostly common in temperate and boreal zones of the NH, they also occur in some localized areas in the humid tropics, and in temperate and cold mountainous regions of the SH (Hawker et al. 1992). The studied podzols of the SH have similar patterns of Hg distribution as for NH podzols (Peña-Rodríguez et al. 2014; Gómez-Armesto et al. 2020), with low pH (4.9 to 6), high organic matter content on top layers and high aluminium and iron distributions.

When comparing the distribution of Hg concentrations in the top five centimeters of soils between different continents, the overall Hg burden of tropical forests is about ten-fold that of the temperate and boreal forests of the NH (Roulet et al. 1999; Amirbahman and Fernandez 2012; Wang et al. 2019) (Table 2; Fig. 2). The origin of this Hg has been attributed to two sources: (1) lithogenic origin with accumulation of natural Hg by ferralitic pedogenesis (Roulet et al. 1998) and (2) atmospheric origin (from natural or anthropogenic sources) particularly through litterfall deposition (Fostier et al. 2015).

### Mercury in African, Australian and Asian soils

The diversity of biomes (e.g. rainforests, forests, shrublands, grasslands and deserts) in tropical Africa and Asia is likely to also reflect a large variability in the natural Hg cycle (Fig. 2). In Africa, a few studies on Hg in background soils in Tanzanian oxisols suggest that ion exchange may be involved in Hg translocation and retention (Semu et al. 1986, 1987, 1989); however, these studies are limited to the analysis of the theoretical soil adsorption capacity under laboratory conditions and neither consider the in situ accumulation of Hg nor compare Hg concentrations in the different horizons. Background Hg concentrations measured in situ in Africa ranged between 13 and 48 ng g^−1^ in soils around northern Lake Victoria (East Africa) (Campbell et al. 2003); between 15 and 50 ng g^−1^ in sediments of the Berg River (South Africa) (Kading et al. 2009); and below 20 ng g^−1^ in soils collected around Lake Chilwa (Malawi) (Mussa et al. 2020). Mercury concentrations in more remote soils in Dar es Salaam (Tanzania) were also low (below 15 ng g^−1^) (Nipen et al. 2022).

While East Africa may have low Hg in soils, this may not be the same for tropical West Africa, where soil types are more comparable to those of South America. Background Hg concentrations in soils from Ghana (West Africa) ranged from below the detection limit (3 ng g^−1^) to 190 ng g^−1^ (Rajaee et al. 2015), a similar Hg concentration range to that of the Tapajos region in South America. Thus, although little is known about the distribution of Hg in soils of West Africa, it is likely that they are characterized by elevated Hg concentrations.

Background Hg concentrations in soils of the Australian continent are lower than those in South America (Fig. 2) (Jardine and Bunn 2010; Lintern et al. 2020; Schneider 2021), reported at 30 ± 18 ng g^−1^ (de Caritat and Cooper 2011; Reimann and de Caritat 2017). Atmospheric measurements from inland Australia also hint at low Hg concentrations in ackground soils, with atmospheric Hg^0^ much lower at these sites (0.6 – 0.7 ng m^−3^) (Howard and Edwards 2018; MacSween et al. 2020) than at other SH sites (~ 1 ng m^−3^) (Slemr et al. 2015; Sprovieri et al. 2016), possibly reflecting a combination of low soil Hg, low anthropogenic emissions and the fact that other SH observations are primarily from coastal sites with largely oceanic influence.

Despite the low Hg concentration in Australian soils (Fig. 2), background soils have been suggested as the dominant Hg emission source to the atmosphere in Australia, responsible for 70% of total emissions (Nelson et al. 2012). The relative importance of soil emission is due to the low anthropogenic Hg emission in the country (Nelson et al. 2012) and lack of Hg^0^ exchange with the NH due to the short atmospheric Hg lifetime of 3–6 months (Horowitz et al. 2017; Saiz-Lopez et al. 2020; Shah et al. 2021; Zhang and Zhang 2022). Recent investigations suggest that soil emissions may in fact be balanced by dry deposition and surface uptake at local scales (Fisher and Nelson 2020; MacSween et al. 2020).

There is no firm hypothesis for the disparity in Hg concentrations between SH continents, but it is believed that the African and Australian continents have experienced a long and complex history of weathering under previous wet climatic conditions (prior to aridization) (Pillans et al. 2005; Gonzalez-Alvarez et al. 2016). In contrast to the Hg-rich soils of the Amazon described above, the low Hg concentrations in eastern and southern African and Australian soils (Fig. 2, Table S1) likely reflect the prolonged and deep chemical weathering of the regolith in these regions, leaching Hg from the soils. These anomalies support the notion that Hg may have been leached from the soils on the African continent, and likely too from soils on the Australian continent. The lower soil Hg concentrations in the SH regions also influence atmospheric Hg concentrations.

The few studies in tropical regions of Asia indicate lower Hg concentrations than soils of South America. Mean soil concentrations at background locations were typically below 100 ng g^−1^ (Zarcinas et al. 2004a, b). Thus, there is preliminary evidence that Hg levels in soils in the SH and tropics are higher in South America and West Africa compared to East Africa and Southeast Asia.

### Mercury in soils under volcanic influence

Mercury concentrations in soils of volcanic areas in the SH are within the concentration ranges found in volcanic soils of the NH (3–640 ng g^−1^) (Peña-Rodríguez et al. 2012). In Patagonia, the SH region where Hg in volcanic soils has been most studied (Fig. 2), Hg concentrations vary according to volcanic sources. For instance, topsoil under the influence of the Copahue volcano has an average Hg concentration of 370 ± 160 ng g^−1^ (Pérez Catán et al. 2020), while soils under the influence of the Puyehue Cordón Caulle volcanic complex have a topsoil Hg concentration of 75 ± 34 ng g^−1^) (Diéguez et al. 2022). This demonstrates the importance of local studies on individual volcanic Hg contribution.

## Mercury deposition via litterfall and throughfall

Direct uptake of atmospheric gaseous Hg^0^ by plants may constitute the largest global mechanism for removal of atmospheric Hg (Wang et al. 2016; Jiskra et al. 2018; Obrist et al. 2021; Zhou and Obrist 2021). The Hg is transferred to soils as litterfall when plants die off and shed leaves, as well as via throughfall when rain washes deposited Hg off the plant surfaces. As such, litterfall and throughfall have been used as proxies to measure Hg dry deposition to terrestrial environments (Fostier et al. 2000). Isotopic analyses indicate that 50–80% of vegetation and soil Hg derives from atmospheric Hg^0^ uptake by plants, with the remaining from wet deposition and aerosol deposition (Demers et al. 2013; Jiskra et al. 2015; Enrico et al. 2016; Obrist et al. 2017).

In the SH and tropics, forests are mainly evergreen (Allaby 2010), with the exception of the temperate rainforests in southern South America and Australasia (east and southeast Australia, Tasmania, New Zealand). Studies of Hg^0^ uptake in the SH have focused almost exclusively on the tropics, and research in the unique temperate environments of the SH is needed to understand the Hg^0^ processes in this area.

### The high mercury uptake capability of tropical rainforests

**Fig. 3:**
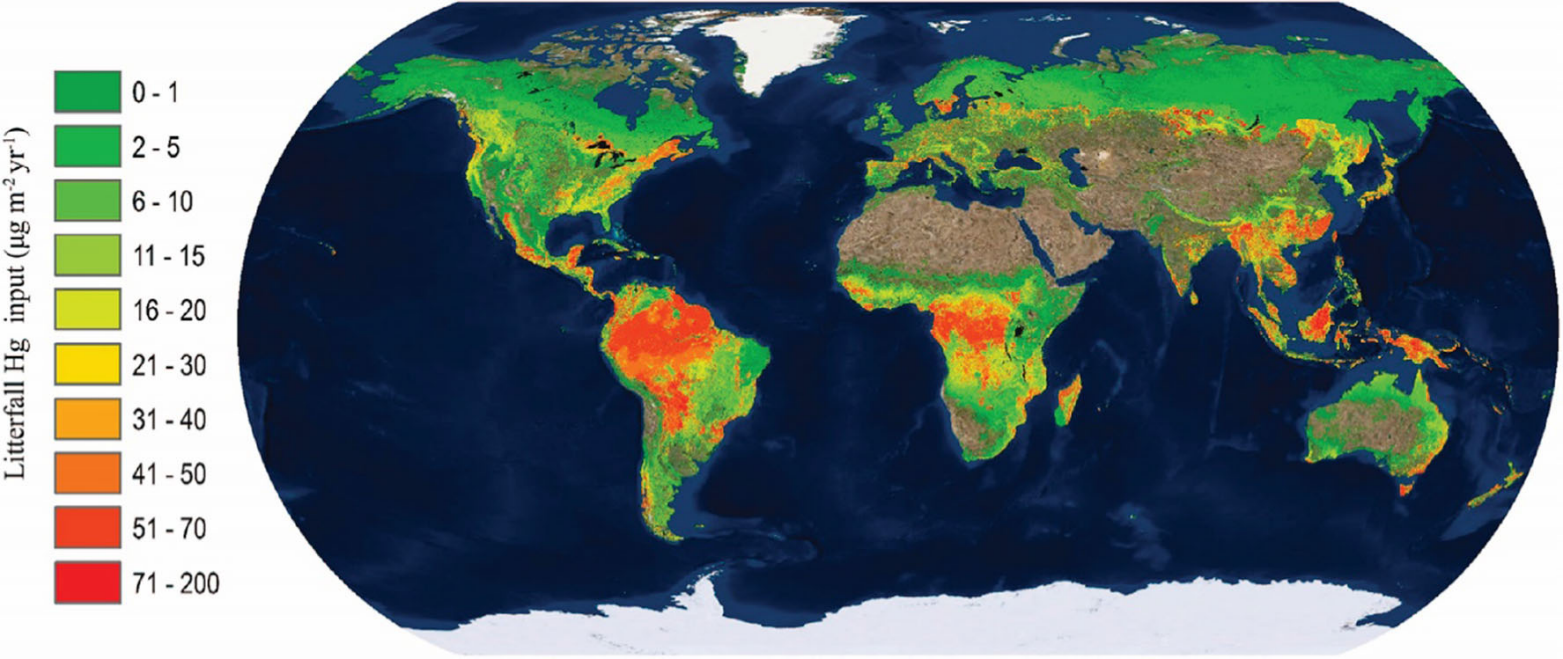
Gridded Hg deposition through litterfall from observed and modelled fluxes. Reprinted from Wang et al. 2016, Assessment of Global Mercury Deposition through Litterfall, Environmental Science & Technology 50: 8548–8557, Copyright 2016, with permission from Elsevier

The tropics are the regions with the highest litterfall fluxes in the world (Fig. 3) (Wang et al. 2016). The deposition flux decreases with latitude, with 70% of total deposition in the tropical/subtropical regions and 30% in the temperate/boreal regions (Wang et al. 2016). Central Africa and the Amazon Basin are the regions with the highest estimated atmospheric Hg^0^ removal through litterfall in the world, with an average Hg uptake of 65.0 ± 30.0 g km^−2^ yr^−1^ (Wang et al. 2016). This higher deposition in the tropics is a result of environmental, physical and biological factors including solar irradiation, photo-reduction, air temperature, altitude, plant species, leaf age, leaf placement and growing season (St. Louis et al. 2001; Ericksen and Gustin 2004; Stamenkovic and Gustin 2009a; Laacouri et al. 2013; Obrist et al. 2017).

The Hg uptake capacity in evergreen tropical forests is likely favoured by two factors: the large leaf area index (LAI) and the longer leaf lifespan (the time at which the leaves from a cohort emerged until they dropped) (Wohlgemuth et al. 2020; Feinberg et al. 2022). Plants with higher LAI and longer leaf lifespan have a much larger capacity to uptake Hg^0^. This is because, as for CO_2_ assimilation, the seasonality in Hg^0^ uptake by vegetation is mainly driven by stomatal processes (Jiskra et al. 2018; Yuan et al. 2019; Zhou and Obrist 2021), which are dependent on leaf anatomy (Stamenkovic and Gustin 2009b; Schneider et al. 2019).

The longer leaf lifespan in tropical forests also favours Hg uptake and higher Hg concentrations in tropical forest vegetation (Pleijel et al. 2021). For example, when compared with leaves of conifers and deciduous tree species in the boreal NH (Sweden), leaves of tropical evergreen trees from a montane rainforest in Rwanda were found to have two-fold higher Hg concentrations at the end of the growing season (21.5 ng g^−1^ vs. 9.2 ng g^−1^) due to the lack of seasonal abscission and longer leaf life span (Pleijel et al. 2021). Tree species of the temperate humid forests of Andean Patagonia (39°-45°S, 71°W) also show higher Hg concentrations in leaves of evergreen species (*Nothofagus dombeyi,* average 38.8 ng g^−1^) than deciduous species (*Nothofagus antarctica,* average 25.2 ng g^−1^) (Juárez et al. 2016).

The highest mean Hg concentration in vegetation has been reported in the Amazon (53 ± 24 ng g^−1^), where it is almost twice the global average Hg concentration in vegetation (34 ± 21 ng g^−1^) (Zhou et al. 2021). At the same time, atmospheric Hg^0^ concentrations are lower in the central Amazon than elsewhere in Brazil (Sprovieri et al. 2016; Quant et al. 2021). Both phenomena are probably due to the efficiency of the Amazonian rainforest in trapping atmospheric Hg^0^ (Jiskra et al. 2018), likely in combination with Hg removal as air moves inland across the continent (similar to Hg processes reported in the NH) (Gustin et al. 2020, Write et al., 2014). The high Hg concentrations in leaves combined to the high net primary productivity of the evergreen broadleaf tropical forests driven by leaf production (Stephenson and van Mantgem 2005; Zhou and Obrist 2021) is a possible explanation of the higher Hg flux in the Amazon when compared to other ecosystems (Fostier et al. 2015). Furthermore, in evergreen forests, epiphytes (bromeliaceae, orchids, ferns, lichens and moss) have the potential to assimilate ~ 12% of the total atmospheric Hg^0^ (Zhou and Obrist 2021), thereby further enhancing Hg^0^ uptake in tropical rainforests.

### Low atmospheric mercury in the Southern Hemisphere: the role of vegetation mercury uptake

A prominent interhemispheric gradient exists for atmospheric Hg concentrations, with lower Hg concentrations in the tropics and in the SH mid latitudes than in the NH (Sprovieri et al. 2016). Defined as ΔHg = [Hg0]|_45°N_–[Hg0]|_45°S_, the average interhemispheric gradient is 0.52 ng m^−3^ and is largest in February and smallest in September (0.68 ng m^−3^ and 0.36 ng m^−3^, respectively) (Jiskra et al. 2018). Although the lower atmospheric Hg^0^ concentrations in the SH can be explained in part by lower anthropogenic emissions and less land area (Jiskra et al. 2018), the high Hg^0^ removal capacity of the tropical evergreen forests and other forested SH ecosystems may also play a significant role. The year-round litterfall flux of evergreen forests may explain the absence of seasonal variation of Hg^0^ concentration in the SH. A recent study has also shown that model estimates of Hg^0^ uptake based on data from temperate NH forests underestimated observed fluxes measured in the Amazon (Feinberg et al. 2022). Integrating model processes that increase Hg^0^ uptake in tropical forests, such as higher LAI, resulted in better agreement between observed and modelled Hg^0^ dry deposition velocity and atmospheric Hg^0^ concentrations and seasonal variations, both in the tropical and in the midlatitude locations (Feinberg et al. 2022).

## Oceanic mercury sources and sink

The differences in land and ocean area between hemispheres (along with the larger anthropogenic inputs into the NH) influence the exchange of Hg between the atmosphere and the ocean. The SH is 81% ocean vs. 60% in the NH, and 57% of the global ocean is in the SH. However, the differences between atmospheric deposition to the ocean and oceanic evasion of Hg^0^ to the atmosphere are smaller than would be expected from the ocean area alone. The evasion of Hg^0^ from the SH ocean has been estimated as 2343 Mg yr^−1^ compared to 1468 Mg yr^−1^ for the NH (Huang and Zhang, 2021). Similarly, total ocean deposition of ionic Hg (Hg^II^) is higher in the SH than in the NH (2536 vs. 1659 Mg yr^−1^). The current generation of models indicate that the ocean is a net sink for atmospheric Hg.

Lake and peat core analyses have been used to argue that natural background emissions (prior to enhanced anthropogenic inputs) were higher in the SH than in the NH due to its larger ocean area (Li et al. 2020). However, it should be noted that most of the SH long-term monitoring sites used are strongly ocean-influenced (coastal or island sites) and therefore may not be fully representative of the overall SH background (Table 1). Regardless, the hemispheric difference in the terrestrial/ocean area influences both the current and pre-industrial Hg^0^ concentrations in the atmosphere due to the different source/sink relationships between the atmosphere and ocean vs. atmosphere and land.

While there are few observational studies of either dissolved Hg^0^ in surface waters or Hg^0^ air-sea exchange in the SH ocean, research cruises provide evidence for the ocean being a net Hg source to the atmosphere in both the subtropical South Pacific gyre (Bowman et al. 2016; Mason et al. 2017) and the South Atlantic (Bratkič et al. 2016). In contrast to the observations, global modelling predicts that the SH ocean is a net sink for atmospheric Hg. Although there may be locations and seasonal periods where the ocean is a net source to the atmosphere, on average, models suggest that Hg^II^ deposition to the SH ocean is greater than ocean evasion (Zhang et al. 2019; Huang and Zhang, 2021). This may be due to the fact that the uptake of Hg^0^ by the ocean has been underestimated in the past, as has been suggested by isotopic analysis of samples from various oceans (Jiskra et al. 2021). Evasion estimates are based on few observations collected on research cruises (discussed above), and there are no long-term datasets to allow for more accurate predictions of net ocean Hg^0^ evasion. However, given the larger ocean area and lower anthropogenic atmospheric inputs in the SH, the importance of ocean evasion as a source of Hg^0^ to the boundary layer of the atmosphere is higher for the SH (> 50%) than for the NH (mostly < 50%) (Zhang et al. 2019).

**Fig. 4:**
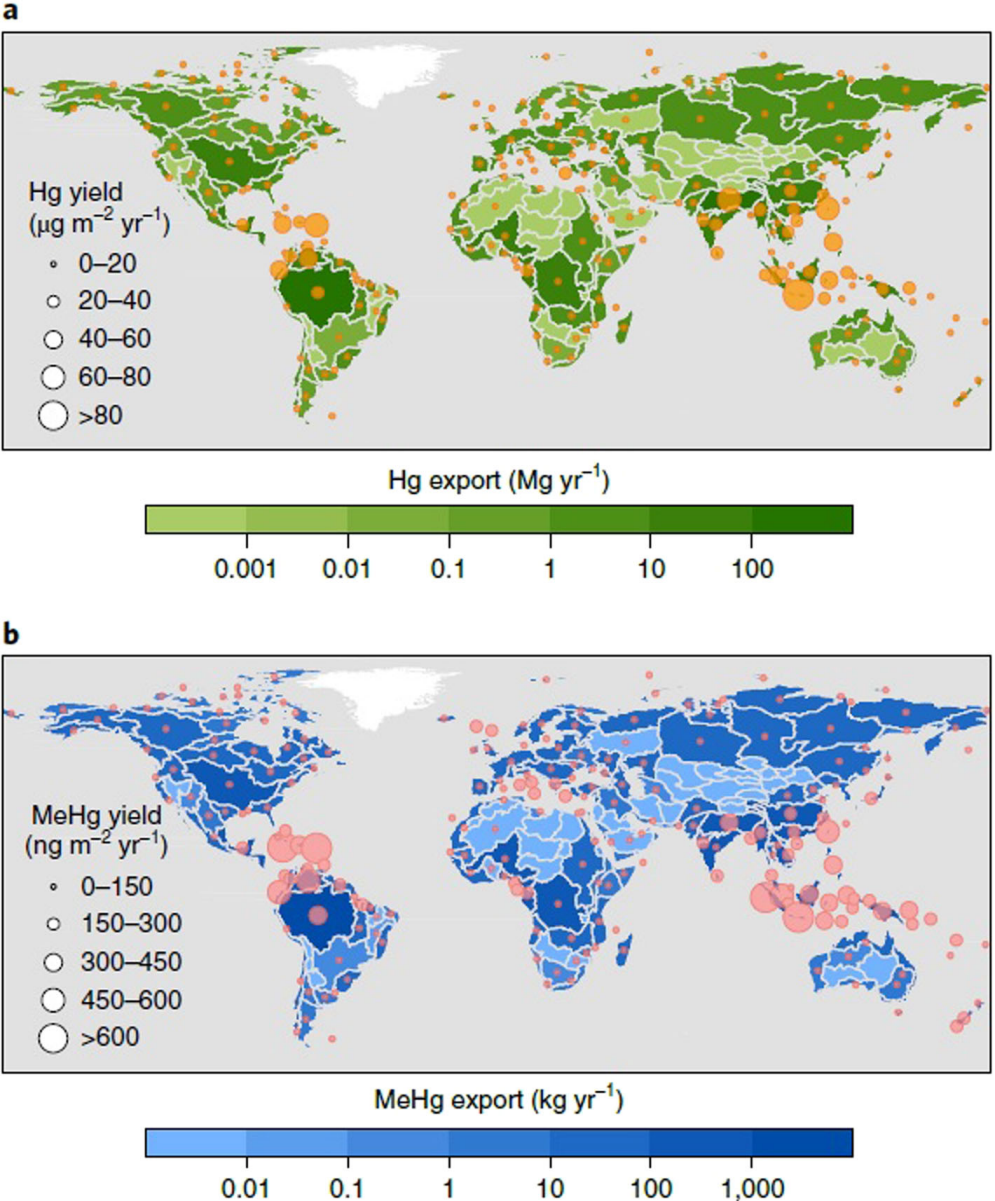
Yields of riverine mercury exports in global river basins. Yields of riverine Hg export (flux divided by drainage area) from different river basins: (a) total Hg and (b) MeHg. Reprinted by permission from Springer Nature: Nature Geosciences vol. 14. Reprinted from Liu, M.; Zhang, Q.; Maavara. T; Liu, S.; Wang, S.; Raymond, P.A. Rivers as the largest source of mercury to coastal oceans worldwide, pp. 672–677. Copyright 2021

In terms of external inputs to the ocean, riverine inputs are concentrated in the NH, with roughly a third of the total riverine inputs being into the SH ocean (Liu et al. 2021). This ratio is comparable with the distribution of land, as a third of the total land is in the SH. The hemispheric distribution of riverine inputs is even more different for the temperate regions (Fig. 4, Table 3). Temperate NH (20–60°N) rivers account for 30–40% of the global riverine Hg inputs, and this is mainly driven by the high Hg inputs from Asia (Fig. 4). In contrast, temperate SH (20–60°S) river inputs only account for 2.5% of the global riverine Hg inputs (Fig. 4). This coincides with the overall arid nature of land in the SH midlatitudes.

**Table 3:** Percentage of total riverine THg and MeHg that is in each latitude band, given as mean (min–max). From Liu et al. (2021)

Latitude	% of global riverine THg input [mean (min–max)]	% of global riverine MeHg input [mean (min–max)]
60–90° N	4% (3–4%)	6% (5–6%)
20–60° N	37% (30–40%)	29% (26–31%)
0–20° N	31% (25–40%)	32% (25–44%)
0–20° S	26% (14–40%)	30% (14–42%)
20–60° S	3% (2–5%)	4% (2–6%)
60–90° S	0%	0%

In the tropics, Hg inputs are more comparable: depending on the dataset used, somewhere between 25 and 40% of the total global riverine inputs are in the tropics in each hemisphere (Liu et al. 2021). Methylmercury (MeHg) inputs to the ocean from rivers are also mostly significant in low latitude areas (Fig. 4, Table 3), with ~ 80% of the global flux for both Hg and MeHg (813 and 7.6 Mg yr^−1^, respectively) occurring in the tropical zone from 30° N to 30° S (Liu et al. 2021). Thus, the total combined contribution of atmospheric deposition and riverine inputs to Hg in the SH open ocean is lower than in the NH. Interestingly, Hg concentrations in SH surface waters are not significantly lower than found in the NH (Bowman et al. 2020), even though external inputs of Hg are lower. This may reflect the overall lower primary productivity of the SH, and therefore the lower removal of Hg via settling of particles from the surface ocean, although this could be an artifact of the limited comprehensive Hg data available for the SH oceans.

## Mercury methylation processes

Mercury methylation seems to be ecosystem-specific, with complex interactions between environmental factors that favour Hg methylation, creating either synergistic or antagonistic effects on methylation rates (Ullrich et al. 2001; Bravo and Cosio 2020). Current understanding of Hg methylation in the SH is largely based on research in the aquatic bodies and flooded soils of the tropics and Amazon, with only a few exceptions. In temperate Australia (Tasmania), two studies of Hg and MeHg concentrations and processes in surface lake reservoir waters, sediments and soils point to high methylation rates in upland catchments (Bowles et al. 2003a, b). Mercury methylation studies in Africa are also limited. Where available, such studies report a lack of correlation between MeHg and organic matter (Lusilao-Makiese et al. 2014; Kgabi and Ambushe 2021; Tulasi et al. 2021). The dry nature and unique features of the African environment warrant further studies on the significant role these factors in Hg methylation on the continent.

### Wetlands as mercury methylation hotspots

**Fig. 5:**
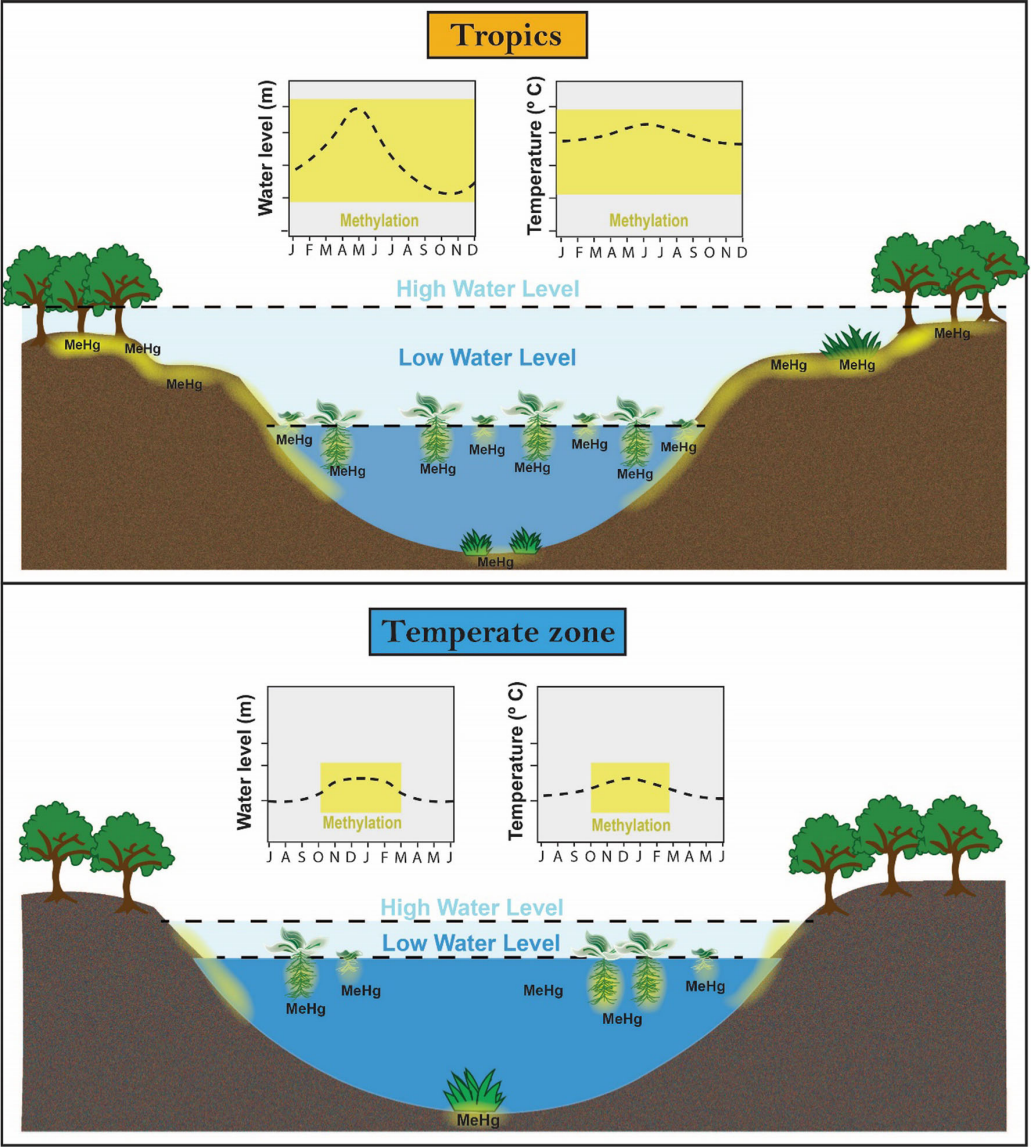
Schematic view of Hg methylation hotspots (highlighted in yellow) in aquatic zones in the tropics vs. the temperate zone. In the tropics, methylation hotspots occur in the flooded forest and in the periphyton on submerged surfaces of macrophytes. In temperate zones, methylation hotspots occur in sediments, epilithon and epiphyton. Note that in the temperate zones, Hg methylation rates are at a minimum in winter. Letters at the bottom of the graphics refer to the months of the year

Wetlands and lake sediments are important hotspots to Hg methylation as these systems commonly have physical, chemical and biological conditions that promote methylation (Ullrich et al. 2001; Selin 2009; Branfireun et al. 2020). While wetlands and lakes rich in macrophytes and periphyton are important Hg methylation hotspots in the NH (Hamelin et al. 2015; Gentès et al. 2017; Leclerc et al. 2021), in equatorial South America, wetlands are characterized by high Hg methylation rates that occur in large floating meadows (Fig. 5) (Guimarães et al. 2000a, b; Roulet et al. 2000). These meadows develop from floating and submerged macrophytes that flourish under favourable physical and chemical conditions in the tropics (e.g. high temperatures year-round) (Guimarães et al. 1998).

In the tropics, the large flood pulses that take place annually play an important role in Hg methylation (Fig. 5) (Lázaro et al. 2016). In the Rio Madeira of the Amazon Basin, for instance, water levels increase up to 14 m yr^−1^, creating unique microhabitats on vast stretches of flooded land that provide ideal grounds for Hg methylation (Guimarães et al. 2000a, b; Roulet et al. 2000). Enhanced Hg methylation rates were also reported during anaerobic decomposition of terrestrial organic matter following episodic flooding events in Lake Murray, Papua New Guinea (Bowles et al. 2002). This is similar to observations in the Amazon and points to large flood pulses being a common phenomenon of tropical systems that enhance Hg methylation. However, more studies are needed to better understand the link between such flood pulses and Hg methylation in tropical systems, particularly in Southeast Asia and tropical Africa, where information on such processes is scant.

The increased Hg methylation in periphyton microhabitats and long trophic chains in the tropics contribute to increased Hg bioaccumulation and biomagnification in food chains of these systems (Bowles et al. 2001; Molina et al. 2010; Azevedo-Silva et al. 2016; Nyholt et al. 2022). Methylmercury concentrations in Lake Murray, Papua New Guinea, showed increased bioaccumulation and biomagnification with trophic level, with the proportion of MeHg increasing from < 1% in plants to 94% in piscivorous fish (Bowles et al. 2001). In remote areas of the Amazon (far from Hg point sources), fish have high Hg concentrations (up to 17.6 µg g^−1^ dry weight), which have been attributed to high rates of trophic magnification and high Hg concentrations lower in the food chain (Nyholt et al. 2022).

In ocean waters, most MeHg is likely produced in situ rather than transported offshore from rivers, with MeHg having a short lifetime in the surface ocean (Liu et al. 2021). As a result, MeHg concentrations in surface ocean waters of the SH are not significantly lower than in the NH, except when compared to specific locations such as the Mediterranean Sea and the northwestern Pacific Ocean, where (compared to other oceans) high MeHg concentrations in the water column and in tuna fish have been recorded (Mason et al. 2012; Tseng et al. 2021; Médieu et al. 2022).

## Geogenic sources

**Fig. 6:**
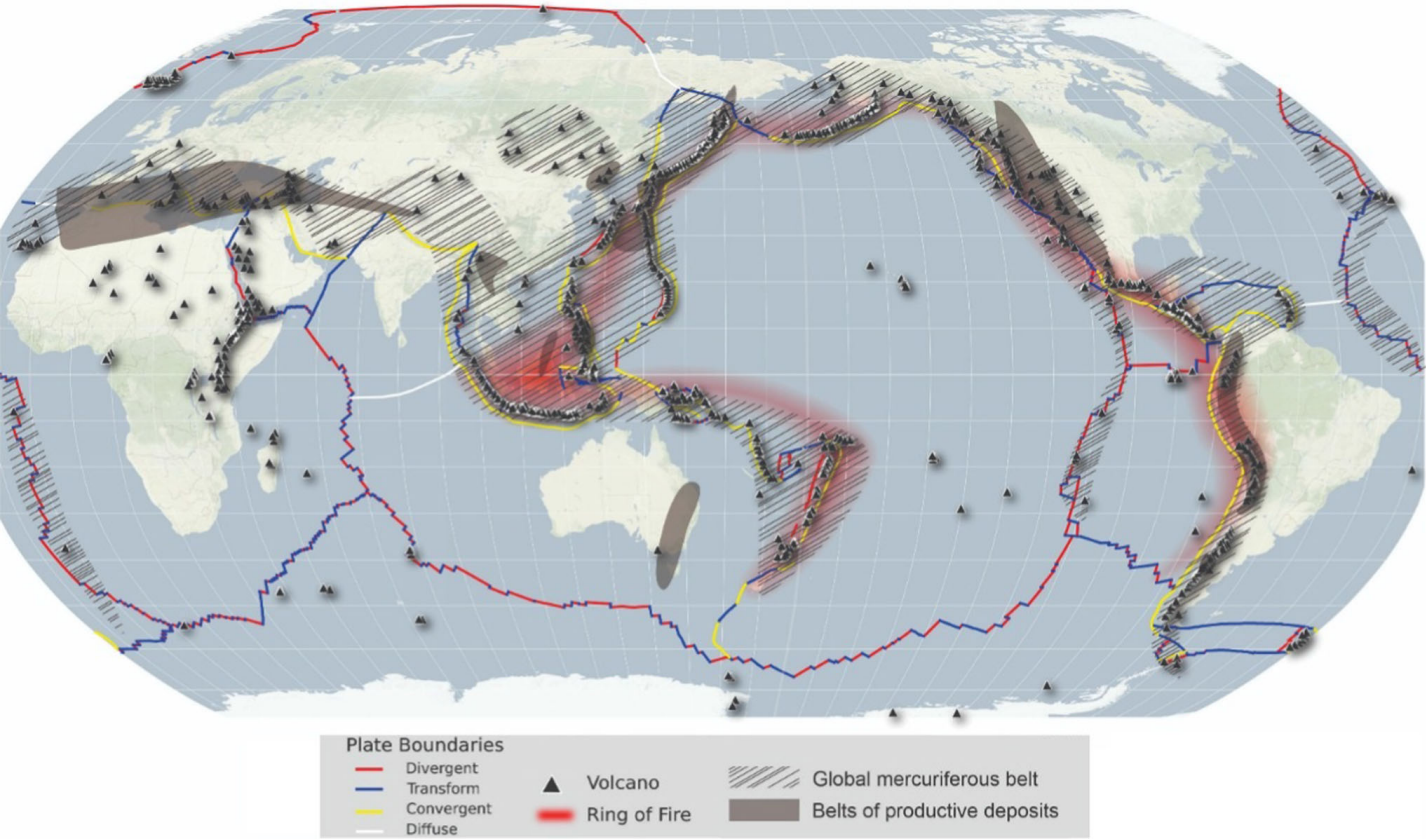
Global distribution of volcanoes, tectonic plates, mercuriferous belts and mercury productive deposits. Image combines information from USGS, (Bailey et al. 1973; Gustin et al. 1999). Map background by CartoDB and Stamen Design, under CC BY 3.0. Data by OpenStreetMap, under ODbL. Map projection: Robinson

Geogenic and volcanic Hg sources are extensive in the SH. They may be considerably more important in SH environments than in the NH as they occur in areas with few other Hg sources (Fig. 6). The most active volcanoes in the SH belong to the Circum-Pacific Belt (Ring of Fire), which includes major active volcanic arcs and oceanic trenches. In the SH, the main volcanic areas are the Peru–Chile, Java (Sunda), Bouganville, Tonga and Kermadec trench (Kirianov 2007). Of lesser influence but with significant active volcanoes are the East African Rift System, the Cameroon Volcanic Line in West Africa and the Indian Ocean Islands (Lenhardt and Oppenheimer 2014).

### Volcanic mercury emissions

In western South America, the Andean Volcanic Belt is an important Hg source, extending through Ecuador, Colombia, Peru, Bolivia, Chile and Argentina (Fig. 6). It is divided into four zones (Northern, Central, Southern and Austral Volcanic Zones) comprising volcanoes diverse in activity, morphology and products (Stern 2004; Bagnato et al. 2011). The limited data available on environmental Hg concentrations along the Andean Volcanic Belt show a strong influence of Hg volcanic emissions on Hg atmospheric concentrations in this area (Edwards et al. 2021).

Elevated atmospheric Hg^0^ concentrations were measured in Los Andes Portillo, in Chile, during the eruption of the Puyehue volcano in June 2011. Mercury concentrations of 10 ng m^−3^ were measured 50 km downwind from this volcanic area, while measurerements in San Carlos de Bariloche (background area) were recorded as 2 ng m^−3^ for the same period (Higueras et al. 2014). New measurements in Chacaltaya (Bolivian Andes) have shown mean Hg^0^ concentrations of 0.89 ± 0.01 ng m^−3^, with higher Hg concentrations occuring when the site was under the influence of air masses from northern Amazonia (0.94 ± 0.02 ng m^−3^) and southern Altiplanic air masses that have passed through degassing volcanoes of the Central Volcanic Zone (1.08 ± 0.08 ng m^−3^) (Koenig et al. 2021). In the southern Andes, atmospheric Hg measurements at the EMMA GMOS Station in Bariloche showed enhanced atmospheric Hg^0^ concentrations (annual mean = 0.86 ± 0.16 ng m^−3^) and changes in Hg speciation patterns associated with winds from volcanic sources (Diéguez et al. 2019).

From the few Hg^0^ measurements in the SH, it is difficult to identify hemispherical differences related to geogenic sources of Hg. Thus far, Hg^0^ concentrations and emissions recorded for the SH are within the same range as for the NH, and the main hemispherical difference is likely to be reflected in the environmental processes Hg undergoes once deposited in the environment. Mercury emissions from volcanic eruptions and geothermal activity in the Andean Volcanic Belt are linked to the high background Hg concentrations in soils of the region (> 100 ng g^−1^) (Ribeiro Guevara et al. 2010; Daga et al. 2016). Similar Hg enrichment has also been recorded in lake sediments of Patagonia, resulting in a significant Hg input to aquatic environments (Soto Cárdenas et al. 2018; Perez Catán et al. 2020; Diéguez et al., 2022) (Fig. 2). The Hg cycle in these Hg-rich terrestrial and aquatic environments of the SH have unique features, described in the previous sections of this paper on background Hg in soils and Hg methylation.

Volcanic degassing and other geothermal sources also play an important role on Hg-rich environments of New Zealand, where natural emissions have been estimated to be on par with anthropogenic emissions (Chrystall and Rumsby 2019). Recent measurements using passive samplers found Hg^0^ concentrations in New Zealand of 11 ng m^−3^ along a fault zone and 50 ng m^−3^ at a geothermal site, significantly higher than measured elsewhere in the world with the exception of a contaminated mining site (Szponar et al. 2020).

### Mercury mineral belts in the SH

Mercury mineral belts are generally concentrated in geological settings associated with previously or currently active tectonic margins, volcanism or geothermal activity (Gustin et al. 2006). One proxy for the amount of Hg in mineral belts is the historical viability of commercial Hg mining. Although the SH has plenty of tectonic and volcanic activities, most of the world’s major Hg mines have been in the NH, and only Peru has important Hg deposits that made viable the commercialisation of Hg as a commodity (McQueen 2011). In Australia, attempts were made to mine mercury between 1869 and 1945 when there was high local Hg demand, but the production was insufficient for commercialisation (McQueen 2011), implying limited recoverable Hg in mineral belts. In New Zealand, where tectonic plates are still active and volcanism is occurring, the abundance of recoverable mineral Hg is also low due to the mafic nature of the volcanics (McQueen 2011), suggesting that volcanism and mineral Hg abundance are not closely linked in the SH.

In Australia, a Hg mineral belt has been reported in the literature as running along the eastern coast all the way to Tasmania (Rytuba 2003). This belt is in agreement with the sites where cinnabar had been mined or prospected in Queensland, New South Wales and Victoria (McQueen 2011). However, soil Hg measurements in Australia do not support the belt being as extensive as reported by Rytuba (2003) (Figs. 2 and 6), and there is still significant uncertainty regarding the influence of geogenic sources in this region. The unsuccessful attempts made in Australia to mine Hg reinforce the likely smaller Hg belt in the country.

The only known measurement of Hg emission fluxes in mercuriferous substrates in the SH is in Australia (Edwards and Howard 2013). Measurements of Hg^0^ showed high Hg emission fluxes (14 ± 1 ng m^−2^ h^−1^ to 113 ± 6 ng m^−2^ h^−1^), significantly higher than non-Hg mineral enriched background sites (0.36 ± 0.06 ng m^−2^ h^−1^) (Edwards and Howard 2013). Overall, the Australian mercuriferous and background data (Edwards and Howard 2013) imply similar processes as seen in NH data (Gustin et al. 1999; Lindberg et al. 1999; Edwards et al. 2005; Schroeder et al. 2005), with naturally enriched substrates showing a strong relationship between emission flux and substrate concentration. However, the environmental factors (particularly temperature) have been shown to be different in Australia, where an increase in temperature as small as 1.2 °C results in an approximately 30% increase in Hg emissions to the atmosphere from naturally enriched sources (Edwards and Howard 2013). This result is illustrative of the limitations of extrapolating NH data to predict Hg emissions in SH environments.

## Conclusions and future research needs

In this synthesis paper, we identifed and described five natural differences between the hemispheres that have implications for Hg cycling. The differences in biogeochemical processes determined by unique geographic and environmental factors demonstrate the need for local research in the SH to understand local Hg processes, releases and emissions.

The range of biomes in the SH calls for more studies on the mechanism of Hg storage by different type of soils, including Hg variation with depth. In particular, there is a lack of information on the pool of Hg moving from terrestrial environments to water bodies. Information regarding the properties, pedogenesis and Hg content of soils in the SH is also needed to provide insights to compare with similar soils of the NH.

A clear trend identified in our literature review is the limited research on natural Hg processes in the SH, which contrasts strongly to the large number of studies motivated by anthropogenic activities. While studies have examined Hg concentrations around contaminated soils (from ASGM sites, for example), there is little information available from remote background sites, which is necessary to fully understand natural Hg processes in the SH. This includes the role of temperature, vegetation type, soil moisture, organic matter and diversity of methylation sites on the Hg cycle. The gaps in data and knowledge are highest in the tropical forests of Africa and Southeast Asia. From the limited published literature, it is clear that there is a wide range of soil Hg concentrations in the different biomes of the SH. It is therefore evident that establishing a threshold limit of Hg concentrations to identify non-contaminated sites in the SH is likely a complex and site-specific task.

Atmospheric Hg^0^ measurements are very sparse in the SH, with results indicating that atmospheric Hg^0^ concentrations in much of the SH are lower than in the NH. This is likely atributted to a combination of low soil Hg, low anthropogenic Hg emissions and a short Hg atmospheric lifetime (~ 3–6 months) hindering interhemispheric exchange. As most Hg^0^ observations in the SH are primarily from coastal sites that have a large oceanic influence, any regional or interhemispheric extrapolations of the biogeochemical cycling of Hg are best reserved until a broader scope of atmospheric Hg^0^ measurements are published for the SH.

The Hg cycle in the Patagonian region has unique features not yet explored. Dust (mainly from volcanism) is a source of Hg with the potential for long-range transport to Antarctica by westerly winds. No information is known about Hg transport from Patagonian dust and whether the dust accelerates snow and ice melt in Antarctica, and whether it could in turn enhance the release of Hg locked in the cryosphere, as reported by others (Stephen et al. 2021). Furthermore, the reduction of continental ice fields in Patagonia due to warming and drying trends in southern South America could impact Hg cycling, although the magnitude of any such effect is completely unknown. Glacial melting may release large quantities of Hg locked in ice into the atmosphere and downstream ecosystems and is an important topic for future Hg studies in the region.

Most studies on Hg uptake by vegetation in the SH and tropics are limited to the Amazon. More studies that measure Hg uptake and concentrations in other SH vegetation types and climates are needed to fully understand the mechanism for atmospheric Hg^0^ trapping by vegetation and the global importance of this sink. This is particularly the case for Patagonia, Africa and Southeast Asia. The unique environments of evergreen mixed forests (evergreen broadleaf hardwoods and conifers) of the SH are also expected to impact Hg^0^ vegetation uptake processes. Data on these processes would provide crucial direction on the present-day vegetation sink and the implications of deforestation for the global Hg cycle.

For oceans, there are little data on Hg speciation and the processes controlling Hg and MeHg in the SH and are further proposed as a focus of future Hg studies. While Hg modelling and limited stable isotope Hg analyses have given insights into the importance of the ocean in Hg cycling in the SH, there is a need to obtain more data on ocean Hg concentrations and speciation in the SH region. The literature review also revealed more studies on Hg dynamics around Antarctica compared to the other larger oceans of the SH. Upcoming cruises through the GEOTRACES Programme and other initiatives will provide much needed data; however, there is still a need for further Hg research in the vast open oceans of the SH. In particular, high-resolution measurements of Hg speciation in the marine atmosphere and of surface water Hg^0^ concentrations are needed to better quantify air–sea exchange dynamics and how they may change in the future. While there are some Hg measurements in southern Africa, these are not sufficient to quantify this flux accurately on a regional and hemispheric scale. Furthermore, there is a dearth of studies of Hg measurements and concentrations in temperate rivers of the SH, with most studies focused on the larger tropical rivers. An in-depth review of Hg processes in the Antarctic is needed to determine the contribution of this unique continent to the biogeochemical cycling of Hg in the SH.

Most studies of Hg methylation in the SH have focused on aquatic bodies of equatorial South America. Research in equatorial Africa and Southeast Asia is needed to fully understand the role of flood pulse processes and high biodiversity in enhancing Hg methylation and biomagnification up food chains in the SH. Measurements of MeHg in the arid environments of southern Africa, Australia and southern South America are needed as these will provide new insights on Hg methylation processes in dry environments that experience the impacts of global change.

Arc volcanism prevails in the SH, and the majority of the most active volcanic regions have been understudied. Limited measurements from the Andes and New Zealand indicate that volcanic and geothermal sources can have a very large local impact on atmospheric Hg^0^. However, important Hg measurement gaps exist in continental and oceanic arc systems, including the Andean Volcanic Belt, the Aleutian Arc, Indonesia, the Vanuatu arc, New Guinea and the Solomon Islands. The Papua New Guinea and Indonesian regions in particular have complex tectonics and are very active seismically, but no Hg data are available. Currently, estimates of volcanic Hg fluxes are mostly derived from spatially and temporally limited data and, in many cases, with a high level of uncertainty due to methodological constraints. The lack of data from geologically active zones makes it difficult to estimate the natural fluxes of Hg to the atmosphere and the impacts on terrestrial and aquatic systems.

## Supplementary Information

Below is the link to the electronic supplementary material.

Supplementary file1 (XLSX 91 KB)
